# Zero-contrast cardiac resynchronization therapy device implantation in heart failure patients with renal impairment

**DOI:** 10.1016/j.hroo.2026.02.026

**Published:** 2026-03-06

**Authors:** Biagio Sassone, Marco Zuin, Angelo Melpignano, Martina De Raffele, Francesca Tiberia, Michele Malagù, Santo Virzì, Francesco Vitali, Cristina Balla, Enrico Bertagnin, Matteo Bertini

**Affiliations:** 1 Department of Translational Medicine, University of Ferrara, Ferrara, Italy; 2 Cardiology Unit, Department of Cardio-Thoraco-Vascular Sciences, AUSL Ferrara, Cento, Ferrara, Italy; 3 Department of Cardio-Thoraco-Vascular Sciences and Public Health, University of Padova, Padova, Italy; 4 Department of Cardiology, Padova South Hospitals, Monselice, Padova, Italy; 5 Cardiology Unit, Sant’Anna University Hospital, University of Ferrara, Ferrara, Italy

**Keywords:** Cardiac resynchronization therapy, Chronic kidney disease, Contrast-induced nephropathy, Left ventricular lead, Death, Heart failure hospitalization

## Abstract

**Background:**

Coronary sinus venography is commonly used during cardiac resynchronization therapy (CRT) to guide left ventricular (LV) lead placement; however, iodinated contrast poses a substantial risk of contrast-induced acute kidney injury in patients with chronic kidney disease (CKD). Evidence supporting contrast-sparing CRT techniques remains limited.

**Objective:**

This study aimed to assess the feasibility, safety, and renal outcomes of a zero-contrast CRT implantation strategy in patients with CKD.

**Methods:**

Consecutive patients with CKD (estimated glomerular filtration rate [eGFR] <60 mL/min/1.73 m^2^) undergoing CRT at 2 centers between January 2023 and June 2025 were analyzed. Procedures were initiated using a contrast-free approach, with coronary sinus venography reserved as a bailout strategy. Predictors of ≥20% eGFR decline were evaluated using multivariable logistic regression. Secondary outcomes included an increase in an LV ejection fraction of ≥5 percentage points at 3–6 months and 1-year all-cause mortality and heart failure hospitalization.

**Results:**

Overall, 113 patients were analyzed. Zero-contrast implantation was successful in 57.5% of cases. Renal function remained stable after zero-contrast implantation (change in eGFR +3.6 mL/min/1.73 m^2^; *P* = .08), whereas it declined significantly in the contrast group (−7.9 mL/min/1.73 m^2^; *P* < .001). Postprocedural acute kidney injury occurred in 1.5% vs 14.6% of patients, respectively (*P* = .004). Contrast use (odds ratio 6.8, 95% confidence interval 1.5–30.9; *P* = .013), lower baseline eGFR, and diabetes independently predicted a ≥20% decline in eGFR. Complication rates, LV ejection fraction improvement, and 1-year mortality or heart failure hospitalization were similar between groups.

**Conclusion:**

Zero-contrast CRT implantation is feasible, safe, and effective, with better renal preservation in patients with CKD.

**Key Findings**

▪ In patients with chronic kidney disease undergoing cardiac resynchronization therapy (CRT), a zero-contrast implantation strategy was feasible in 57.5% of cases, with iodinated contrast used as a bailout in the remainder.
▪ Renal function remained stable with zero-contrast CRT, whereas contrast use was associated with significant estimated glomerular filtration rate (eGFR) decline and a higher incidence of acute kidney injury.
▪ Contrast exposure independently predicted a ≥20% eGFR reduction.
▪ Procedural safety and clinical outcomes were comparable between strategies, but even modest contrast use carried a substantial renal risk.

## Introduction

Cardiac resynchronization therapy (CRT) is an established and highly effective treatment for drug-refractory heart failure with a wide QRS.^1^, ^2^, ^3^ The therapeutic benefit of CRT depends on optimal left ventricular (LV) lead positioning, which is typically guided by coronary sinus (CS) venography using iodinated contrast.

However, CS venography requires iodinated contrast administration, which carries a well-recognized risk of contrast-induced acute kidney injury (CI-AKI).^4^ Although the impact of postprocedural renal impairment has been extensively evaluated in patients undergoing percutaneous coronary intervention,^5^ data specifically addressing CRT are limited. Worsening renal function has been reported in up to 15% of patients after CRT implantation, particularly among those with preexisting chronic kidney disease (CKD), and is directly associated with adverse clinical outcome.^6^, ^7^, ^8^, ^9^

To address this evidence gap and better define the renal implications of iodinated contrast use during CRT in patients with CKD, we evaluated the feasibility, safety, and efficacy of a zero-contrast transvenous LV lead implantation strategy in this high-risk population.

## Methods

### Patient selection

We reviewed both clinical and procedural data from all consecutive adult patients referred for CRT implantation, with or without defibrillator capability, at 2 tertiary cardiology centers: the Cardiology Unit of SS.ma Annunziata Hospital, Cento, Ferrara, Italy, and the Cardiology Unit of the University Hospital Sant’Anna, Ferrara, Italy. The cohort was collected from the Contemporary Cardiac Stimulation in Clinical Practice: Left, Biventricular, Right, and Conduction System Pacing registry (NCT06324682) and included both de novo implants and device upgrades from conventional pacemakers or implantable cardioverter-defibrillators, performed between January 2023 and June 2025. The study protocol was approved by the Area Vasta Emilia Centro Ethics Committee (identifier: 825/2022/Farm/AOUFe) and conducted in accordance with the principles of the Declaration of Helsinki. A written informed consent was obtained from all patients before the implantation procedure.

The inclusion criteria were (1) age >18 years, (2) a guideline-based indication for CRT according to the current European guidelines,^1^ and (3) a baseline estimated glomerular filtration rate (eGFR) of <60 mL/min/1.73 m^2^ upon admission.

Conversely, the exclusion criteria included (1) a documented history of iodine allergy contraindicating iodinated contrast media; (2) a previous failed attempt at CRT implantation with documented inability to access the CS from the intended venous route; (3) hemodynamic instability at the time of the index procedure, precluding a prolonged or stepwise implantation strategy; and (4) noncardiac comorbidities associated with an expected life expectancy of <1 year. Patients who declined to provide a written informed consent were also excluded. In addition, patients in whom transvenous LV lead implantation was unsuccessful were not considered in the present analysis.

### Technique for LV lead placement

**Figure 1:**
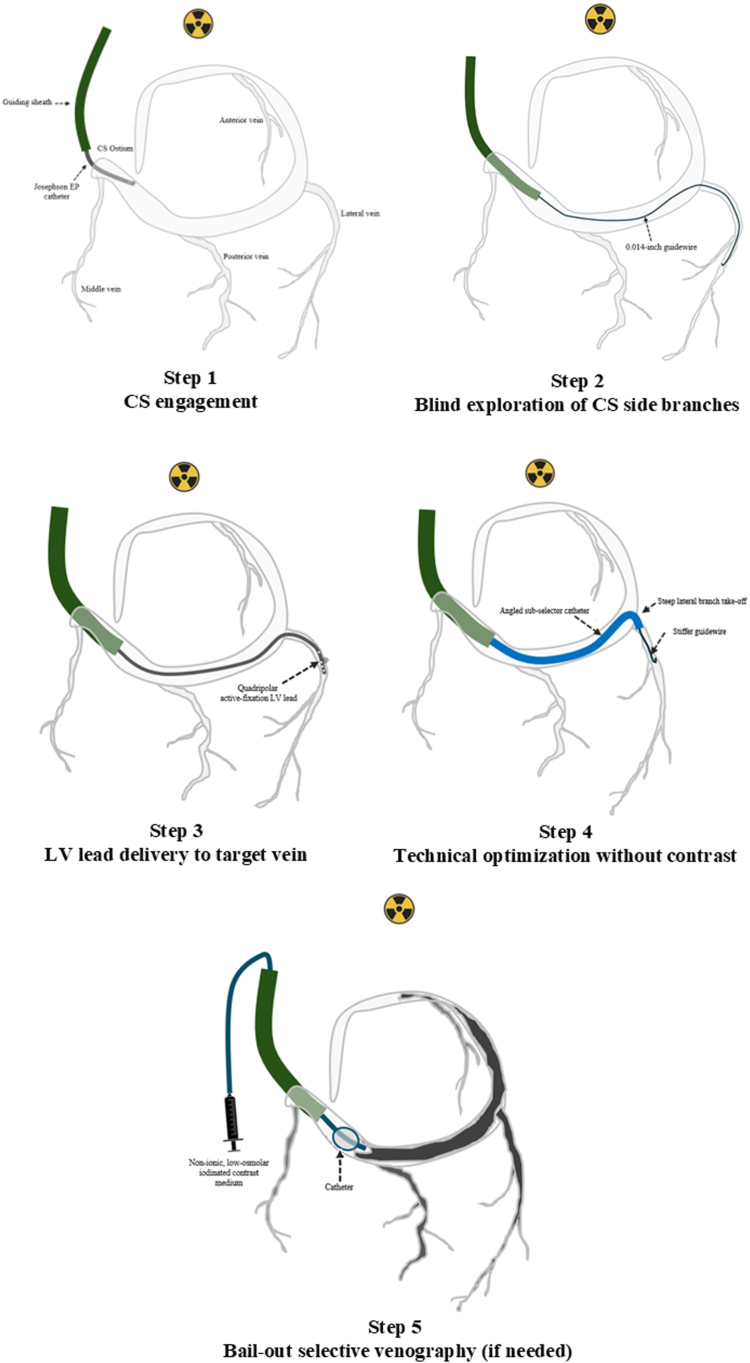
Schematic representation of a stepwise zero-contrast strategy for LV lead implantation during CRT. An initial nonangiographic approach is systematically attempted, reserving selective venography as a bailout option only after failure of blind side-branch cannulation. Step 1: CS access is obtained using a preshaped guiding sheath, with a Josephson-curved EP mapping catheter used for orientation; CS positioning is then confirmed in orthogonal fluoroscopic projections (LAO/RAO). Step 2: after removal of the EP mapping catheter, a 0.014-in soft guidewire with a curved tip is introduced for nonangiographic exploration of CS side branches; the guidewire is advanced distally under fluoroscopic guidance to delineate the vessel course. Step 3: the preferred target is a lateral or posterolateral vein, avoiding distal or apical positions whenever possible; the LV lead is advanced over the guidewire into the selected branch, followed by electrical and clinical assessment, including pacing threshold, lead stability, and evaluation for phrenic nerve stimulation. Step 4: in case of challenging access, adjunctive techniques may be used, including angled subselector catheters, the buddy-wire technique, or escalation to a stiffer guidewire; if the target vein proves unsuitable, the LV lead is repositioned into an alternative CS branch. Step 5: selective venography is performed only after unsuccessful nonangiographic exploration (bailout approach), using contrast as a final anatomic roadmap; contrast administration is minimized to the lowest feasible volume. CRT = cardiac resynchronization therapy; CS = coronary sinus; EP = electrophysiology; LAO = left anterior oblique; LV = left ventricular; RAO = right anterior oblique.

LV transvenous epicardial lead implantation was performed via the coronary venous system under fluoroscopic guidance using a contrast-sparing, stepwise approach. Access to the CS was obtained through standard venous access using dedicated guiding sheaths with different preshaped curves, selected according to operator preference and individual cardiac anatomy. To facilitate orientation and selective engagement of the CS ostium without contrast injection, a Josephson-curved electrophysiology mapping catheter was advanced through the guiding sheath and used as a steerable probe. A schematic overview of the stepwise zero-contrast implantation strategy is presented in Figure 1. Once stable CS engagement was achieved, the mapping catheter was withdrawn and the distal tip of the guiding sheath was positioned at or just inside the CS ostium. Correct positioning was confirmed using orthogonal fluoroscopic projections (30° left anterior oblique and 30° right anterior oblique views).

After CS access, an initial attempt to identify and cannulate suitable CS side branches was performed without venography using a 0.014-in soft, curved-tip guidewire. Under fluoroscopic guidance, the guidewire was gently advanced and manipulated to probe potential venous tributaries, allowing indirect delineation of the vessel course based on wire trajectory and movement. Once a side branch was successfully engaged, the guidewire was advanced distally to provide support, and the LV lead was delivered over the wire to the predefined target region (Supplemental Video 1).

The preferred target veins were lateral or posterolateral CS branches, and successful LV lead placement was defined as a final position within a lateral or posterolateral myocardial segment according to the classification proposed by Singh et al.^10^ When multiple suitable branches were available, selection prioritized the most lateral position, with preference for basal or midventricular segments and avoidance of distal or apical locations. If the initially selected branch was electrically unsuitable, anatomically unstable, or associated with phrenic nerve stimulation, alternative branches were explored, aiming to position the lead as close as possible to the intended target region.

When blind guidewire probing was unsuccessful, particularly in the presence of steep branch take-off angles, telescoping techniques were used. In particular, angled inner subselector catheters were advanced over the guidewire through the guiding sheath to facilitate selective cannulation of side branches. Once the guidewire was positioned distally within the tributary, the inner catheter was withdrawn, and the LV lead was advanced over the wire. In cases of tortuous venous anatomy or insufficient support for lead advancement, additional strategies included the use of a stiffer 0.014-in guidewire and/or placement of a parallel “buddy wire” to improve rail support and facilitate lead delivery.

No predefined time limit was imposed for the contrast-free exploration phase. The decision to terminate the zero-contrast attempt was left to the operator’s discretion when suitable venous branches could not be identified or when the accessed vein was deemed unsuitable owing to anatomic instability, suboptimal electrical parameters, or phrenic nerve stimulation. In such cases, selective CS venography was performed as a bailout strategy to obtain an anatomic roadmap of the CS and its tributaries, and the procedure was completed according to standard venography-guided implantation techniques.^11^

Quadripolar LV leads were used in all patients, predominantly active-fixation models (Attain Stability Quad™, Medtronic), to enhance lead stability and reduce the risk of dislodgement in the absence of contrast-defined venous anatomy. All procedures were performed by 2 experienced, high-volume operators (B.S. and M.B.) with more than 10 years of experience in cardiac implantable electronic device implantation and more than 400 CRT procedures each.

### Periprocedural renal protection protocol

All patients with CKD underwent a standardized renal protection protocol to reduce the risk of CI-AKI. Iodinated contrast for other diagnostic procedures (eg, computed tomography or angiography) was avoided for at least 72 hours before and after CRT implantation. Potentially nephrotoxic medications, including nonsteroidal anti-inflammatory drugs and selected antibiotics, were withheld 24 hours before and 48–72 hours after the procedure whenever feasible.^12^, ^13^, ^14^ Intravenous hydration with isotonic saline (0.9% sodium chloride) was administered according to LV ejection fraction (LVEF): 1 mL/kg/hour for an LVEF of ≥40% and 0.5 mL/kg/hour for an LVEF of <40%, starting 6–12 hours before the procedure and continued 6–24 hours after the procedure.

When CS venography was required as a bailout option after an initial zero-contrast attempt, a nonionic, low-osmolar iodinated contrast medium (Iopamiro®, Bracco Imaging) was used at the lowest feasible volume. These measures, combined with the zero-contrast strategy whenever feasible, were designed to preserve renal function and minimize CI-AKI risk.

### Variables and definitions

In accordance with Kidney Disease: Improving Global Outcomes (KDIGO) guidelines,^15^ baseline CKD was defined in patients with a documented history of renal dysfunction and an eGFR of <60 mL/min/1.73 m^2^ on admission. This degree of renal impairment represents the strongest predictor for contrast-induced nephropathy (CIN) in patients receiving iodinated contrast media and aligns with the CIN Consensus Working Panel.^16^ Acute kidney injury (AKI) was defined according to KDIGO criteria as an increase in serum creatinine of ≥50% from baseline within 7 days.^17^ eGFR was calculated using the CKD-EPI equation, which incorporates age and sex and provides a more accurate assessment of renal function than serum creatinine alone.^18^ In all patients, eGFR was performed 24 hours before and 48–72 hours after the procedure using an enzymatic method traceable to the isotope-dilution mass spectrometry reference standard.^19^

Within 4 weeks before and 3–6 months after CRT implantation, patients underwent a comprehensive echocardiographic evaluation (Philips EPIQ CVx 3-dimensional cardiology ultrasound system, Koninklijke Philips N.V., Eindhoven, The Netherlands), including measurement of LVEF in accordance with the American Society of Echocardiography and European Association of Echocardiography guidelines.^20^

Before hospital discharge, CRT devices were programmed according to guideline-recommended procedures to ensure optimal biventricular pacing.^21^

Postimplant follow-up information was obtained through record linkage between the regional electronic hospital discharge database (International Classification of Diseases, Ninth Revision, coded) and the regional mortality registry, using each patient’s unique pseudonymized identifier. Additional clinical data were collected during scheduled outpatient visits and using device-specific remote monitoring systems.

### Outcomes

The primary outcome of the study was renal function change, assessed as the absolute and relative change in eGFR from before to after the procedure. Both AKI and/or a ≥20% decline in eGFR were considered clinically relevant endpoints. A relative decline in an eGFR of ≥20% was selected a priori as a clinically meaningful threshold, given that changes of this magnitude exceed expected biological and laboratory variability and have been associated with adverse renal outcomes in CKD populations, even when not meeting formal KDIGO AKI criteria.^22^^,^^23^

Secondary outcomes included absolute increase in an LVEF of ≥5 percentage points at 3–6 months, 1-year all-cause mortality, and heart failure hospitalization (HFH).

### Statistical analysis

Continuous variables were expressed as mean ± standard deviation or median (interquartile range), as appropriate, and compared using the Student *t* test or the Wilcoxon rank-sum test. Categorical variables were summarized as counts (percentages) and compared using χ^2^ or Fisher’s exact test. Between-group comparisons were performed using the Student *t* test or the Mann–Whitney U test for continuous variables and χ^2^ or Fisher’s exact test for categorical variables. Absolute and relative changes in eGFR were calculated and compared between groups. Logistic regression analysis was used to identify predictors of ≥20% eGFR decline, including iodinated contrast use, age, baseline eGFR, diabetes, heart failure severity (New York Heart Association [NYHA] III–IV vs I–II), LVEF, and procedure type (de novo vs upgrade). Odds ratios (ORs) with 95% confidence intervals (CIs) were reported. Multivariable linear regression was also performed to evaluate independent predictors of postprocedural eGFR change. To address potential model overfitting owing to the limited number of patients exposed to contrast, we performed a sensitivity analysis using a reduced multivariable logistic regression model. This reduced model included only the strongest univariable predictors of postprocedural ≥20% eGFR decline: contrast use, baseline eGFR, and diabetes.

All analyses were conducted using Stata version 16 (StataCorp, College Station, TX) and SPSS version 20.0 (IBM Corp, Armonk, NY). A 2-sided *P* < .05 was considered statistically significant.

## Results

### General characteristics

During the study period, 171 patients were referred for CRT. A transvenous LV lead could not be implanted in 2 cases: in 1, uncontrollable phrenic nerve stimulation at the available venous side branches prevented stable lead positioning; in the other, no accessible lateral branches of the CS could be visualized during venography in the setting of a persistent left superior vena cava. Therefore, these 2 patients were excluded from the present analysis. Among the remaining 169 patients who underwent successful transvenous CRT, 113 (67%) met the criteria for CKD and were included in the analysis. The zero-contrast strategy was successful in 65 patients (57.5%), whereas conventional CS venography was subsequently required in the remaining 48 patients. Baseline demographics and clinical characteristics are presented in Table 1. Age, sex, BSA, prevalence of atrial fibrillation, ischemic cardiomyopathy, previous myocardial infarction, revascularization procedures, LVEF, and NYHA functional class were similar between the 2 groups.

**Table 1:** Comparison of chronic kidney disease patients undergoing cardiac resynchronization therapy implantation with or without iodinated contrast use for coronary sinus angiography

Variable	Zero-contrast group(n = 65)	Contrast group(n = 48)	*P* value
Age, y, mean ± SD	79.0 ± 8.9	77.4 ± 8.0	.34
Men, n (%)	45 (69.2)	33 (68.7)	.95
BSA, m^2^, mean ± SD	1.89 ± 0.24	1.90 ± 0.20	.86
Creatinine, mg/dL, mean ± SD	1.82 ± 0.2	1.26 ± 0.21	<.001
Preprocedural eGFR, mL/min per 1.73 m^2^, mean ± SD	35.5 ± 10.7	51.1 ± 6.7	<.001
Postprocedural eGFR, mL/min per 1.73 m^2^, mean ± SD	39.1 ± 12.7∗	43.2 ± 8.4†	.54
Diabetes, n (%)	32 (49.2)	13 (27.0)	.01
Atrial fibrillation, n (%)	22 (33.8)	20 (41.6)	.39
Ischemic cardiomyopathy, n (%)	42 (64.6)	23 (47.9)	.07
Previous myocardial infarction, n (%)	34 (52.3)	20 (41.6)	.26
Previous PCI, n (%)	35 (53.8)	19 (39.5)	.13
Previous CABG, n (%)	12 (18.5)	6 (12.5)	.39
LVEF, %, mean ± SD	32.4 ± 6.5	32.4 ± 9.2	.97
NYHA			
I, n (%)	3 (4.6)	4 (8.3)	.63
II, n (%)	40 (61.5)	31 (64.6)	
III, n (%)	20 (30.8)	11 (22.9)	
IV, n (%)	2 (3.1)	2 (4.2)	
De novo implantation, n (%)	53 (81.5)	20 (41.0)	<.001
CRT with defibrillator backup, n (%)	42 (64.6)	31 (64.5)	.99
Procedure time, min, mean ± SD	133.2 ± 40.9	145.1 ± 34.4	.10
Fluoroscopy time, min, mean ± SD	20.5 ± 10.1	24.6 ± 9.1	.03
Fluoroscopy dose, mGy, mean ± SD	529.2 ± 383.9	675.9 ± 543.15	.15
Contrast media, mL, mean ± SD	-	9.6 ± 8.1	-
Procedural complications, n (%)	0	1 (2.0)	2.9
3–6-mo LVESD change, mm, mean ± SD	−5.1 ± 3.7	−5.4 ± 3.9	.72
Relative LVEF change, % of baseline, median (IQR)	+18.4 (1.5–30.7)	+19.0 (5.3–30.7)	.42
1-y all-cause mortality, n (%)	5 (7.7)	2 (4.1)	.43
1-y HFH, n (%)	6 (9.2)	3 (6.2)	.56

BSA = body surface area; CABG = coronary artery bypass grafting; CRT = cardiac resynchronization therapy; eGFR = estimated glomerular filtration rate; LVEF = left ventricular ejection fraction; LVESD = left ventricular end-systolic diameter; HFH = heart failure hospitalization; NYHA = New York Heart Association; PCI = percutaneous coronary intervention; SD = standard deviation.

∗ Comparing pre- and postprocedural eGFR among patients treated without iodinated contrast, *P* = .08.

† Comparing pre- and postprocedural eGFR among patients treated with iodinated contrast, *P* < .001.

Patients undergoing zero-contrast implantation had significantly higher serum creatinine (1.82 ± 0.2 mg/dL vs 1.26 ± 0.21 mg/dL; *P* < .001), lower eGFR at baseline (35.5 ± 10.7 vs 51.1 ± 6.7 mL/min/1.73 m^2^; *P* < .001), and a higher prevalence of diabetes (49.2% vs 27.0%; *P* = .01). De novo CRT implantation was more frequent in the zero-contrast group (81.5% vs 41.0%; *P* < .001).

Procedural characteristics were comparable between the 2 groups, including total procedure duration (133.2 ± 40.9 minutes vs 145.1 ± 34.4 minutes; *P* = .10) and fluoroscopy dose (529.2 ± 383.9 mGy vs 675.9 ± 543.2 mGy; *P* = .15). Conversely, fluoroscopy time was slightly shorter in the zero-contrast cohort (20.5 ± 10.1 minutes vs 24.6 ± 9.1 minutes; *P* = .03). Procedure-related complications occurred in only 1 patient in the contrast group (2.0%), who experienced an accidental puncture of the axillary artery during fluoroscopy-guided venous access without subsequent clinical consequences, whereas no complications were observed in the zero-contrast cohort. The mean total contrast volume in the contrast cohort was 9.6 ± 8.1 mL per patient.

### Primary and secondary outcomes

#### Primary outcome

Renal function remained stable after zero-contrast implantation, with a nonsignificant increase in eGFR from 35.5 ± 10.7 mL/min/1.73 m^2^ to 39.1 ± 12.7 mL/min/1.73 m^2^ (*P* = .08). The mean absolute change in eGFR was +3.6 mL/min/1.73 m^2^, corresponding to a relative increase of 10.1%. In contrast, patients exposed to iodinated contrast experienced a significant decline in eGFR, from 51.1 ± 6.7 mL/min/1.73 m^2^ before the procedure to 43. 2 ± 8.4 mL/min/1.73 m^2^ after the procedure (*P* < .001), representing an absolute decrease of −7.9 mL/min/1.73 m^2^ and a relative decline of 15.5%. Postprocedural AKI occurred in 1 patient (1.5%) in the zero-contrast group and in 7 patients (14.6%) in the contrast group (*P* = .004). In multivariable logistic regression including contrast use, age, diabetes, baseline eGFR, HF severity (NYHA III–IV vs I–II), LVEF, and procedure type (de novo vs upgrade), contrast exposure was independently associated with ≥20% postprocedural eGFR decline (OR 6.8; 95% CI 1.5–30.9; *P* = .013). Lower baseline eGFR (OR 0.92 per mL/min/1.73 m^2^; 95% CI 0.87–0.98; *P* = .006) and diabetes (OR 3.2; 95% CI 1.0–10.1; *P* = .048) were also independent predictors (Table 2). In the reduced multivariate model, contrast use remained strongly associated with postprocedural ≥20% eGFR decline (OR 7.2; 95% CI 2.0–25.8; *P* = .003). Lower baseline eGFR also independently predicted renal deterioration (OR 0.91 per mL/min/1.73 m^2^; 95% CI 0.86–0.97; *P* = .004), as did the presence of diabetes (OR 3.5; 95% CI 1.2–10.3; *P* = .022). These findings were consistent with the results of the full multivariable model, supporting the robustness of the association between contrast exposure and clinically meaningful declines in renal function.

**Table 2:** Multivariable logistic regression for ≥20% decline in eGFR after CRT implantation

Variable	OR	95% CI	*P* value
Contrast use (yes vs no)	6.8	1.5–30.9	.013
Baseline eGFR (per mL/min per 1.73 m^2^)	0.92	0.87–0.98	.006
Diabetes (yes vs no)	3.2	1.0–10.1	.048
Age (per year)	1.03	0.96–1.10	.38
LVEF (per %)	0.97	0.90–1.05	.45
HF severity (NYHA III–IV vs I–II)	1.4	0.4–5.0	.61
Procedure type (upgrade vs de novo)	1.6	0.5–5.3	.42

CI = confidence interval; CRT = cardiac resynchronization therapy; eGFR = estimated glomerular filtration rate; HF = heart failure; LVEF = left ventricular ejection fraction; NYHA = New York Heart Association; OR = odds ratio.

#### Secondary outcome

When response to CRT was defined as an absolute increase in an LVEF of ≥5 percentage points at 6 months, no significant difference was observed between zero-contrast and contrast groups (75.0% vs 71.4%; *P* = .83), and relative LVEF improvement was likewise comparable (median [IQR] 18.4% [1.5–30.7] in the zero-contrast group vs 19.0% [5.3–30.7] in the contrast group; *P* = .42). At 1-year follow-up, all-cause mortality (7.7% vs 4.1%; *P* = .43) and HFH (9.2% vs 6.2%; *P* = .56) were similar between the zero-contrast and contrast groups, indicating comparable midterm clinical outcomes.

## Discussion

Our study provides several important insights. First, complete avoidance of iodinated contrast was feasible and successfully achieved in more than half of the CRT procedures. Second, contrast use was independently associated with a significant postprocedural decline in renal function. Third, the zero-contrast strategy did not compromise procedural safety or midterm clinical outcomes, including 1-year mortality and HFH.

Renal dysfunction is common in real-world CRT populations,^24^ even though patients with advanced CKD were excluded from landmark trials (Table 3). Exposure to iodinated contrast during device implantation may further impair renal function, potentially limiting the clinical benefits of CRT. Despite this concern, evidence supporting contrast-free CRT strategies remains limited. In this regard, the present findings support the use of a zero-contrast approach, which seemed feasible, preserving renal function without adversely affecting procedural success or intermediate-term outcomes in high-risk patients.

**Table 3:** Renal exclusion criteria in the major randomized controlled trials on cardiac resynchronization therapy

Trial	Year of publication	Renal exclusion criteria
MIRACLE	2002	Serum creatinine >3.0 mg/dL
COMPANION	2004	Serum creatinine >2.5 mg/dL (≈eGFR <30 mL/min)
CARE-HF	2005	Creatinine clearance <30 mL/min
REVERSE	2008	Severe CKD excluded (eGFR typically <30–40 mL/min)
MADIT-CRT	2009	End-stage renal disease (on dialysis) excluded
RAFT	2010	eGFR <30 mL/min/1.73 m^2^ (≈7% of screened patients excluded)

CKD = chronic kidney disease; eGFR = estimated glomerular filtration rate.

### Feasibility of zero-contrast CRT

Only 1 small nonrandomized prospective study has evaluated the feasibility of contrast-free CRT,^25^ including 17 patients with an eGFR of <40 mL/min/1.73 m^2^ or iodine allergy. Procedural success was achieved in 16 patients, with no procedure-related complications; however, no data were reported on clinical outcome.

In our study, a complete zero-contrast approach was achievable in 57.5% of patients. The use of contemporary delivery systems, inner subselection catheters, and quadripolar active-fixation leads likely contributed to procedural success and lead stability. Furthermore, the significantly lower proportion of CRT upgrades in the zero-contrast group (*P* < .001) likely reflects the greater technical challenges of these procedures, where preexisting right-sided leads reduce maneuverability and often necessitate earlier contrast use to assess venous patency.

Notably, patients in the zero-contrast cohort had higher baseline serum creatinine and lower eGFR. This likely reflects a selection bias owing to operator awareness of the increased risk of contrast-induced nephrotoxicity in recruited patients. Moreover, it is possible that, because no predefined time limit was set for the initial attempt without contrast, operators may have persisted longer in completing the procedure without contrast in patients with more severe CKD, whereas they may have switched earlier to contrast in others.

Despite this potential bias, total procedural duration, fluoroscopy time, and fluoroscopy dose did not differ significantly between groups, suggesting that the zero-contrast approach does not prolong or compromise procedural efficiency

### Primary and secondary outcomes

Several previous series reported that contrast-related renal injury after CRT implantation is not uncommon and may be substantially under-recognized. In this regard, in a single-center cohort of 68 procedures, Cowburn et al^6^ reported contrast nephropathy in 14% of cases, with a markedly higher risk when baseline creatinine was ≥2.26 mg/dL. In a larger cohort of 822 CRT recipients, Tester et al^7^ found an overall contrast nephropathy incidence of 8%, increasing to 11.8% when ≥95 mL of contrast was administered. More recently, in a subanalysis of the randomized TRUST-CRT population (n = 98) applying contemporary KDIGO criteria,^8^ CI-AKI occurred in 10.2% and was nearly doubled in patients with an eGFR of <60 mL/min per 1.73 m^2^. Moreover, CI-AKI independently predicted higher long-term mortality. Similarly, Strisciuglio et al^9^ reported a 12% CI-AKI rate after CRT, which was associated with lower LVEF recovery and worse survival.

Consistent with these previous observations, in our cohort of patients with CKD, exposure to iodinated contrast was independently associated with a nearly 7-fold higher odds of ≥20% postprocedural eGFR decline and an approximately 10-fold higher incidence of KDIGO-defined AKI than zero-contrast implantation. The greatest susceptibility was observed among patients with lower baseline eGFR and those with diabetes. Given the relatively small number of patients receiving contrast and the limited number of renal events, concerns regarding overfitting of the full multivariable logistic regression model are valid. To address this, we performed a sensitivity analysis using a reduced model including only the strongest univariable predictors: contrast exposure, baseline eGFR, and diabetes. The results were consistent with the primary analysis, with contrast exposure remaining strongly associated with ≥20% postprocedural eGFR decline. These findings reinforce the clinical relevance of contrast avoidance in patients with CKD undergoing CRT implantation and suggest that the observed associations are robust despite the modest sample size. Notably, these associations were observed despite the very low contrast exposure in our “contrast” cohort, given that venography was performed only as a bailout option after an initial contrast-free attempt, resulting in a mean contrast volume of 9.6 ± 8.1 mL per patient. This volume is markedly lower than that reported in previous CRT series: 146 mL in Cowburn et al,^6^ 77 ± 54 mL in Tester et al,^7^ a median of 40 mL (0–155) in TRUST-CRT,^8^ and a median of 16 mL (16–24) in Strisciuglio et al.^9^ Importantly, available evidence suggests that minimizing contrast volume does not compromise the adequacy of LV lead placement,^7^ supporting efforts to reduce contrast exposure whenever feasible. In contrast, the zero-contrast strategy had a neutral effect on renal function, with a modest, favorable trend in eGFR that may partly reflect the periprocedural intravenous hydration protocol applied for CIN prophylaxis. Importantly, the renal benefit observed with the zero-contrast strategy was not achieved at the expense of CRT efficacy. The proportion of responders based on an absolute LVEF increase of ≥5 percentage points at 3–6 months was similar between groups, suggesting that avoiding iodinated contrast does not compromise LV functional recovery.

Finally, although renal preservation did not translate into differences in 1-year mortality or HFH, prevention of postprocedural renal deterioration remains clinically relevant. Multiple large HF cohorts and meta-analyses have shown that even modest worsening of renal function confers a sustained increase in all-cause mortality and HFH.^6^, ^7^, ^8^, ^9^^,^^26^^,^^27^ Within this context, the absence of detectable differences in midterm outcomes in our relatively small, high-risk sample likely reflects limited statistical power and follow-up duration rather than a lack of prognostic importance of avoiding renal injury.

### Limitations

This study has several limitations. Its retrospective design, moderate sample size, and single-region recruitment may limit the generalizability of the findings. Operator discretion in deciding when to transition from a zero-contrast to a contrast-guided approach may have introduced selection bias, particularly in patients perceived to be at a higher risk of contrast-associated nephrotoxicity. In addition, contrast exposure in the “contrast” cohort was intentionally minimized and typically used only as a bailout option; therefore, our study was not designed to evaluate a dose–response relationship between contrast volume and renal injury. Accordingly, we cannot exclude that higher contrast volumes might be associated with a higher incidence of contrast-associated AKI and potentially different clinical outcomes. In addition, renal function was reassessed only in the early postprocedural period; thus, potential long-term trajectories of renal function could not be evaluated. Another important limitation is that LV lead implantation was performed almost exclusively using an active-fixation quadripolar lead (Attain Stability Quad, Medtronic). The unique design and enhanced maneuverability of this lead may have facilitated successful positioning without contrast, potentially limiting the applicability of our results to centers using different lead systems or more traditional delivery tools. Furthermore, all procedures were performed by high-volume operators with substantial CRT expertise, and similar procedural efficiency and success rates may not be reproducible in lower-volume settings. Despite these limitations, the consistency and magnitude of the association between contrast exposure and postprocedural renal decline support the robustness and clinical relevance of our findings.

## Conclusion

Our findings suggest that a contrast-sparing approach to CRT implantation is feasible in a broad range of patients. In our cohort of patients with CKD, a zero-contrast strategy preserved renal function without compromising procedural success or midterm outcomes. Larger studies are needed to confirm these hypothesis-generating findings.

## Disclosures

The authors have no conflicts of interest to disclose.

## Footnotes

Appendix Supplementary data associated with this article can be found in the online version at https://doi.org/10.1016/j.hroo.2026.02.026.
