# Supplementary material for: Zero-contrast cardiac resynchronization therapy device implantation in heart failure patients with renal impairment
Source: Heart Rhythm O2. 2026 Mar 6;7(6):1105–13. doi: 10.1016/j.hroo.2026.02.026 (PMC13307477; doi:10.1016/j.hroo.2026.02.026)
Supplement: Supplementary Video Legend [file mmc1.docx]

Supplemental Video 1. Fluoroscopic view showing blind guidewire navigation within the coronary sinus using a subselection catheter (no contrast). Left bundle branch area pacing catheter and a right-atrial backup catheter are also visible/in place.
